# Effects of different types of acidic nitrogen-enriched biochar on spinach growth, nitrogen use efficiency, and chemical properties of calcareous sandy soil

**DOI:** 10.1186/s12870-026-08149-x

**Published:** 2026-02-02

**Authors:** Amal S. M. Gad El-hak, Abu El-Eyuoon Abu Zied Amin, Muhammad Abdel-Shakour, Abdalrahman G. Algamal, Refaat M. Mahfouz

**Affiliations:** 1 Chemistry Department, Faculty of Science, Assiut University, Assiut, P.O. Box: 71526, Egypt; 2 Soils and Water Department, Faculty of Agriculture, Assiut University, P.O. Box: 71526, Assiut, Egypt; 3 Egyptian Petroleum Research Institute, Cairo, Egypt

**Keywords:** Available nitrate, Available potassium, Chlorophyll, Nitrogen-enriched biochar, Nitrogen use efficiency

## Abstract

**Background:**

Enriching biochar with nitrogen and using it as a slow-release nitrogen fertilizer is a promising strategy to avoid excessive use of chemical nitrogen fertilizers. Therefore, this study investigated the effect of different types of nitrogen-enriched biochar on the soil’s chemical properties, nitrogen use efficiency (NUE), and growth of spinach in calcareous sandy soil. This pot experiment included 9 treatments: control (unamended soil, CK), willow branches biochar (WB), apple of Sodom biochar (ASB), marvel grass biochar (MB), halfa grass biochar (HB), nitrogen-enriched willow branches biochar (NEWB), nitrogen-enriched apple of Sodom (NEASB), nitrogen-enriched marvel grass biochar (NEMB), nitrogen-enriched halfa grass biochar (NEHB). Nitrogen-enriched biochar and biochar were added at a level of 5 g kg^− 1^ soil. This experiment was conducted on the spinach plant.

**Results:**

Soil available nitrate increased significantly with adding NEHB, NEMB, NEWB, and NEASB compared to the control treatment. Adding HB, NEHB, NEMB, NEWB, and NEASB to the soil significantly improved the fresh and dry shoot of the spinach plant compared to the control. Applying HB, NEHB, NEMB, NEWB, and NEASB treatments increased the fresh shoot of the spinach plant over the control by ~ 62%, 245%, 237%, 275%, and 196%, respectively. On the other hand, adding MB, WB, and ASB treatments decreased the fresh shoot of the spinach plant relative to the control by ~ 14%, 3%, and 14%, respectively. Applying different types of nitrogen-enriched biochar significantly improved the NUE of spinach plant compared to the original biochar treatments.

**Conclusions:**

Our results suggest that nitrogen-enriched biochar can be a promising strategy in sustainable agriculture to increase soil nutrient availability, improve spinach growth, and enhance nitrogen use efficiency in spinach. Nitrogen-enriched biochar can serve as an effective and cost-efficient alternative to chemical nitrogen fertilizers.

## Introduction

Cultivating vegetable crops in rural areas of developing countries is economically profitable, helps reduce poverty and unemployment, and encourages farmers to diversify their crops. Vegetables are significant sources of vitamins and minerals for good human health [1]. In recent decades, population growth and dietary changes have significantly increased the demand for leafy vegetables, especially spinach [2]. Spinach (*Spinacia oleracea* L.) is an important vegetable crop grown for its leaves, which are a rich source of chlorophyll, giving them their dark green color, and have high consumer acceptance. It also has a high nutritional value due to its high content of iron and vitamin A, as well as its high content of vitamin C, minerals, riboflavin, and low calories [3].

The rapid global population growth has led to the massive generation of organic wastes, including crop residues, forest residues, municipal waste, sewage sludge, and certain industrial effluents. The accumulation of these wastes in the environment, in the absence of health awareness, may lead to serious damage [4]. Converting organic feedstocks into biochar reduces harmful gas emissions and improves soil fertility, representing a promising strategy for managing organic feedstocks. Biochar is a carbon-rich material that can be produced from a wide range of organic feedstocks, such as crop residues, forest residues, food waste, municipal waste, sewage sludge, animal waste, and manure [5, 6]. Biochar can be referred to as the “black gold” of future agriculture due to its remarkable properties [7]. Various chemical and physical modification methods can be employed to enhance the properties of biochar for environmental applications. The most widely used chemical modification methods include treating biochar with strong acids, bases, salts, and chemical oxidants [6]. The physical and chemical properties of modified biochar greatly depend on the modification techniques, pyrolysis temperature, feedstock types, and the compounds used for modification [8]. The use of non-modified biochar may result in nutrient deficiencies in the soil. Hence, enrichment biochar techniques with nutrients have made biochar-based fertilizers possible, with great potential to enhance soil properties and crop yield. Biochar enrichment with nutrients is an effective way to overcome the limitations of biochar use: deficiency of some nutrients, high application rates, and high production costs [9]. The slow release of nutrients from enriched biochar into the soil makes it an efficient nutrient source, reducing nutrient losses in crops, decreasing fertilizer demand, and mitigating environmental pollution [10]. Biochar can serve as an effective nutrient carrier for producing low-cost, slow-release fertilizer that enhances crop yield and plant growth. Its high organic carbon content, large surface area, extensive microporosity, and diverse functional groups enable it to retain substantial amounts of plant nutrients [11].

Nitrogen is the primary limiting nutrient, after carbon, hydrogen, and oxygen, and it affects photosynthesis, phytohormones, proteomic changes, and the growth and development of plants throughout their lifecycle [12]. Nitrogen in the soil is present in several forms, including ammonium, nitrate, nitrite, dissolved organic nitrogen, and organic nitrogen [13, 14]. Global demand for nitrogen fertilizers is projected to reach 112 million tons to produce food for about 8 billion people. Despite this, more than half of the applied nitrogen fertilizer is lost to the environment, significantly affecting air, water, and soil quality, as well as biodiversity. Nitrogen loss also contributes to greenhouse gas emissions and climate change [15]. Low use efficiency of applied nitrogen fertilizers in soils represents a major problem in irrigated vegetable cultivation [16]. Therefore, the negative effects of chemical nitrogen fertilizers should be mitigated through sustainable agricultural practices, such as the use of nitrogen-enriched biochar, as investigated in this study. Nitrogen fertilizers added to the soil undergo various processes, including mineralization, immobilization, nitrification, leaching, denitrification, volatilization, and plant uptake [17].

The combined application of biochar and chemical fertilizers has long been the conventional strategy for maximizing the benefits of their interaction in soil. Generally, amending soil with biochar improves total porosity, leading to an increase in water-holding capacity and nutrient retention, which in turn enhances nutrient utilization and crop yield. The interaction between biochar and nitrogen in the soil has a significant impact on the concentrations of ammonium and nitrate, highlighting the important role of biochar in enhancing nitrogen use efficiency via increasing the availability of nitrogen to plant uptake [18]. Biochar applications to the soil improve nitrogen use efficiency, which can be attributed to two important mechanisms: First, biochar itself contains nitrogen, which is gradually released into the soil solution. This may contribute to increases in ammonium and nitrate contents. Second, biochar increases nitrogen retention in soil and limits nitrogen loss by enhancing electrostatic adsorption and ammonium retention, due to biochar’s high cation exchange capacity and a large surface area [19]. Moreover, biochar application decreased nitrate leaching in sandy soil under field conditions [20]. The addition of biochar significantly reduced the leaching of ammonium nitrogen from the soil [21]. In soils, the adsorption of nitrate and ammonium ions occurs on the anion and cation exchange sites of the biochar [11]. The ability of biochar to reduce ammonia volatilization and leaching nitrate from the fertilized soils can be improved through modifications and formulation of biochar-based slow-release nitrogen fertilizers [11, 22]. Moreover, using biochar as a soil amendment caused a significant improvement in the plant growth parameters of spinach plants and also mitigated salt stress [23]. Co-application of biochar with nitrogen fertilizer improved soil physicochemical properties, as well as growth, yield, and quality of noodle rice [24], and enhanced the nitrogen use efficiency of maize plants, decreased total nitrogen loss, and increased maize yield. Biochar increased the proportion of inorganic nitrogen supplied by fertilizer in the inorganic nitrogen pool [25]. Biochar generally contributes to the improvement of multiple soil ecosystem services, yet the extent of its effectiveness is strongly influenced by its physicochemical properties, soil characteristics, and climatic conditions [26]. Sufficient fertilizer application enhances plant growth and increases crop yield. On the other hand, the overuse of chemical fertilizers leads to the accumulation of compounds in edible products, which harms human health and causes economic losses [27]. Agricultural systems in the coming decades will be more dependent on adopting agricultural practices that reduce environmental effects, mitigate and adapt to climate change, to enhance crop production sustainability to ensure food security and safety [28]. The novelty of this study lies in producing nitrogen-enriched biochar by sequential treatments with nitric acid and ammonia solution, applied after pyrolysis of different types of biochar. Due to the presence of anion and cation exchange sites on biochar surfaces, biochar enriched with ammonium and nitrate ions was produced. Furthermore, the novelty of this study lies in the fact that most previous studies relied on enrichment with either nitrates or ammonium, but not both. This post-pyrolysis chemical modification markedly increases nitrogen content, resulting in nitrogen-rich biochar with significant potential to enhance soil fertility and promote sustainable agriculture. This study hypothesizes that (1) biochar and acidic nitrogen-enriched biochar effects on soil properties and spinach growth will vary highly with biochar type and (2) Acidic nitrogen-enriched biochar has a more significant effect on improving soil properties and spinach growth than original biochar. Therefore, this study aims to examine the effect of different types of acidic nitrogen-enriched biochar and original biochar on the soil’s chemical properties, nitrogen use efficiency, and the growth of spinach in calcareous sandy soil. The main goal of this study is to produce acidic nitrogen-enriched biochar that can serve as a slow-release fertilizer and simultaneously act as a carrier for ammonium and nitrate.

## Materials and methods

### Materials

Spinach seeds were purchased from an agricultural supply store. Ammonia solution (25%) and nitric acid (68%) were purchased from El Nasr Pharmaceutical Chemicals Company, Egypt.

## Preparation of biochar and nitrogen-enriched biochar

Four types of plants that are locally available were selected and no permission was needed for collecting plant samples. Marvel grass (*Dichanthium annulatum*) and halfa grass (*Desmostachya bipinnata*) were collected from Assiut University, Assiut, Egypt, while Willow branches (*Salix mucronata*) were collected from El-Ghanayem City, Assiut, Egypt, and apple of Sodom (*Calotropis procera*) was collected from Qena Governorate, Egypt. The plants were pyrolyzed separately at 400 °C for four hours. After completing the pyrolysis process, the different types of biochar were ground separately using a stainless-steel grinder through a 1 mm sieve. To prepare nitrogen-enriched biochar, we took a known weight of biochar and mixed it with distilled water at a ratio of 1:10 to form a suspension. First, concentrated nitric acid (68%) was added to the biochar suspension at a level of 74 ml kg^− 1^ biochar with continuous stirring, and the mixture was left for approximately 48 h. Second, concentrated ammonia solution (25%) was added to the mixture at a level of 104 ml kg^− 1^ biochar with continuous stirring, and the mixture was left for approximately 48 h. Finally, concentrated nitric acid was added to the biochar suspension at a level of 91 ml kg^− 1^ biochar with continuous stirring, and the mixture was left in the air to dry. The amount of nitric acid was greater than the amount of ammonia, which is important for obtaining acidic nitrogen-enriched biochar. The pH of the original and acidic nitrogen-enriched biochar was measured in a 1:10 suspension using a pH meter with a glass electrode. Total C, H, N, and S were determined in all original biochar and acidic nitrogen-enriched biochar treatments, using a CHNS elemental analyzer (Elementar Vario EL, Germany). The properties of original biochar and acidic nitrogen-enriched biochar are presented in Table 1.

**Table 1:** Elemental analysis of different types of Biochar and nitrogen-enriched Biochar

	Concentrations of elements (%)				C/*N* ratio	pH (1:10)
	Nitrogen (*N*)	Carbon (C)	Sulfur (S)	Hydrogen (H)		
HB	1.71	72.23	1.87	1.81	42.26	8.45
NEHB	4.62	68.36	0.69	1.80	14.78	5.17
MB	0.93	63.73	1.06	1.56	68.75	8.86
NEMB	4.33	52.00	0.57	1.36	12.02	5.57
WB	1.77	87.59	0.84	2.73	49.51	7.54
NEWB	6.25	71.57	0.69	2.67	11.46	4.42
ASB	0.70	96.68	0.79	1.63	138.31	8.37
NEASB	3.89	88.68	0.59	1.45	22.80	4.52

HB: halfa grass biochar; NEHB: nitrogen-enriched halfa grass biochar; MB: marvel grass biochar; NEMB: nitrogen-enriched marvel grass biochar; WB: willow branches biochar; NEWB: nitrogen-enriched willow branches biochar; ASB: apple of Sodom biochar; NEASB: nitrogen-enriched apple of Sodom biochar

### Pot experiment

The soil used in the pot experiment was collected from the surface soil layer (0–30 cm) obtained from El-Ghorieb farm, Faculty of Agriculture, Assiut University, Assiut, Egypt. The collected soil was air-dried and crushed to pass through a 2 mm sieve before the pot experiment was performed. The soil properties are shown in Table 2. Two kg of air-dried soil was placed in a plastic pot (14 cm height x 11.5 cm base diameter x 15 cm top diameter) to which the nitrogen-enriched biochar and biochar were added at a level of 5 g kg^− 1^ soil. The soil and biochar were mixed thoroughly. The experiment included 9 treatments: control (unamended soil, CK), willow branches biochar (WB), apple of Sodom biochar (ASB), marvel grass biochar (MB), halfa grass biochar (HB), nitrogen-enriched willow branches biochar (NEWB), nitrogen-enriched apple of Sodom biochar (NEASB), nitrogen-enriched marvel grass biochar (NEMB), nitrogen-enriched halfa grass biochar (NEHB). Five spinach (*Spinacia oleracea* L.) seeds were planted in each pot on 14 November 2023 and irrigated with tap water (0.47 dS m^− 1^). This experiment was performed in a completely randomized design with three replications. After 19 days from planting, the spinach plants in each pot were thinned to three plants. The spinach plant was harvested after 63 days from planting. Then, the fresh shoot was weighed for each pot. The spinach shoot was washed with distilled water and oven-dried at 70 °C. Then, the dry shoot was weighed. Soil samples were collected from each pot after harvesting the spinach, air-dried, crushed, sieved, and stored for chemical analysis. This pot experiment was conducted at the Department of Soils and Water, Faculty of Agriculture, Assiut University, Assiut, Egypt.

**Table 2:** Some chemical and physical properties of the soil under study. Data were average ± standard error (SE)

Property	Unit	Value
Sand	g kg^− 1^	922.00 ± 8.48
Silt	g kg^− 1^	62.00 ± 2.82
Clay	g kg^− 1^	16.00 ± 5.65
Texture	-	Sand
O.M	g kg^− 1^	3.57 ± 0.00
CaCO_3_	g kg^− 1^	195.00 ± 4.24
pH (1:1)	-	7.83 ± 0.35
EC (1:1)	dS m^− 1^	0.70 ± 0.05
Available NH_4_^+^	mg kg^− 1^	81.90 ± 0.00
Available NO_3_^−^	mg kg^− 1^	135.45 ± 3.15
Available P	mg kg^− 1^	5.06 ± 0.04
Available K	mmol kg^− 1^	5.06 ± 0.02

OM: organic matter; CaCO_3_: calcium carbonate; EC: electrical conductivity; NH_4_: ammonium; NO_3_: nitrate; P: phosphorus; K: potassium

### Soil analysis

The particle size distribution of the soil under study was determined using the pipette method [29]. Soil calcium carbonate content was determined using the calcimeter method [30]. Soil organic matter is estimated by the dichromate oxidation procedure [31]. Soil pH was measured in a 1:1 (soil: water) suspension using a pH meter with a glass electrode [32]. The electrical conductivity (EC) of soil extracts was measured in a 1:1 (soil: water) extract using an electrical conductivity meter [32]. Available nitrogen in soil samples was extracted using 1 M KCl [33]. The available nitrogen, including ammonium (NH_4_^+^-N) and nitrate (NO_3_^−^-N) in soil extracts, was determined by the Kjeldahl method in two steps: first, the estimation of NH_4_^+^ alone in the soil extracts, and second, the estimation of NO_3_^−^ in the soil extracts through adding Devarda’s alloy to convert the entire NO_3_^−^ into NH_4_^+^ [34]. Available phosphorus (Olsen-P) in the soil samples was extracted by 0.5 M NaHCO_3_ at pH 8.5 [35]. Phosphorus in the extracts was estimated by colorimetric analysis using the chlorostannous phosphomolybdic acid method [32]. Available potassium in soil samples was extracted with 1 M ammonium acetate, pH 7, and then measured by a flame photometer [36].

### Plant analysis

The concentrations of nitrogen, phosphorus, and potassium were estimated in dried shoots of spinach plant after digestion with a method of H_2_SO_4_-H_2_O_2_ [37]. The contents of the digestion tube were quantitatively transferred into a 100-mL volumetric flask. Total nitrogen in all digestive samples was analyzed with the Micro-Kjeldahl method, and phosphorus was measured colorimetrically by the phosphomolybdic acid method in a sulfuric acid system [32]. Potassium in all extracts was measured by flame photometry. Nutrient uptake (mg pot^− 1^) in spinach plant was calculated using the following equation [38].

$$\begin{aligned} &\:\mathrm{N}\mathrm{u}\mathrm{t}\mathrm{r}\mathrm{i}\mathrm{e}\mathrm{n}\mathrm{t}\:\mathrm{u}\mathrm{p}\mathrm{t}\mathrm{a}\mathrm{k}\mathrm{e}\:\left(\mathrm{m}\mathrm{g}\:{\mathrm{p}\mathrm{o}\mathrm{t}}^{-1}\right)\\&=\frac{\mathrm{N}\mathrm{u}\mathrm{t}\mathrm{r}\mathrm{i}\mathrm{e}\mathrm{n}\mathrm{t}\:\mathrm{c}\mathrm{o}\mathrm{n}\mathrm{c}\mathrm{e}\mathrm{n}\mathrm{t}\mathrm{r}\mathrm{a}\mathrm{t}\mathrm{i}\mathrm{o}\mathrm{n}\:\mathrm{i}\mathrm{n}\:\mathrm{d}\mathrm{r}\mathrm{y}\:\mathrm{s}\mathrm{h}\mathrm{o}\mathrm{o}\mathrm{t}\:\left(\mathrm{m}\mathrm{g}\:{\mathrm{k}\mathrm{g}}^{-1}\right)\:\mathrm{X}\:\mathrm{d}\mathrm{r}\mathrm{y}\:\mathrm{s}\mathrm{h}\mathrm{o}\mathrm{o}\mathrm{t}\:\left(\mathrm{g}\:{\mathrm{p}\mathrm{o}\mathrm{t}}^{-1}\right)}{1000} \end{aligned}$$

The calculation of nitrogen use efficiency was according to [39].

$$\begin{aligned} &\:\mathrm{N}\mathrm{i}\mathrm{t}\mathrm{r}\mathrm{o}\mathrm{g}\mathrm{e}\mathrm{n}\:\mathrm{u}\mathrm{s}\mathrm{e}\:\mathrm{e}\mathrm{f}\mathrm{f}\mathrm{i}\mathrm{c}\mathrm{i}\mathrm{e}\mathrm{n}\mathrm{c}\mathrm{y}\:\left(\mathrm{m}\mathrm{g}\:\mathrm{y}\mathrm{i}\mathrm{e}\mathrm{l}\mathrm{d}\:{\mathrm{m}\mathrm{g}}^{-1}\mathrm{N}\:\mathrm{a}\mathrm{p}\mathrm{p}\mathrm{l}\mathrm{i}\mathrm{e}\mathrm{d}\right)\\&=\frac{\mathrm{C}\mathrm{r}\mathrm{o}\mathrm{p}\:\mathrm{y}\mathrm{i}\mathrm{e}\mathrm{l}\mathrm{d}\:\mathrm{w}\mathrm{i}\mathrm{t}\mathrm{h}\:\mathrm{a}\mathrm{p}\mathrm{p}\mathrm{l}\mathrm{i}\mathrm{e}\mathrm{d}\:\mathrm{n}\mathrm{i}\mathrm{t}\mathrm{r}\mathrm{o}\mathrm{g}\mathrm{e}\mathrm{n}\:\left(\mathrm{m}\mathrm{g}\:{\mathrm{p}\mathrm{o}\mathrm{t}}^{-1}\right)}{\mathrm{A}\mathrm{m}\mathrm{o}\mathrm{u}\mathrm{n}\mathrm{t}\:\mathrm{o}\mathrm{f}\:\mathrm{n}\mathrm{i}\mathrm{t}\mathrm{r}\mathrm{o}\mathrm{g}\mathrm{e}\mathrm{n}\:\mathrm{a}\mathrm{p}\mathrm{p}\mathrm{l}\mathrm{i}\mathrm{e}\mathrm{d}\:\left(\mathrm{m}\mathrm{g}\:{\mathrm{p}\mathrm{o}\mathrm{t}}^{-1}\right)} \end{aligned}$$

The amount of nitrogen applied is expressed as the amount of nitrogen present in biochar and nitrogen-enriched biochar.

### Statistical analysis

The data obtained in this study were statistically analyzed using MSTAT-C software (version 2.10). Statistically significant differences between treatments were performed by Tukey’s honestly significant difference (Tukey’s HSD) test at a probability level of 0.01(*p* ≤ 0.01).

## Results

### Characteristics of original biochar and nitrogen-enriched biochar

The results obtained from this study indicated that the type of feedstock played an important role in influencing the chemical characteristics of biochar. The elemental composition of different original biochar types produced at 400 °C is tabulated in Table 1. Nitrogen content was 1.71%, 0.93%, 1.77%, and 0.70% for HB, MB, WB, and ASB, respectively. The carbon content was 72.23%, 63.73%, 87.59%, and 96.68% for HB, MB, WB, and ASB, respectively. The sulfur content was 1.87%, 1.06%, 0.84%, and 0.79% for HB, MB, WB, and ASB, respectively. Hydrogen content was 1.81%, 1.56%, 2.73%, and 1.63% for HB, MB, WB, and ASB, respectively. The elemental composition of different types of nitrogen-enriched biochar is tabulated in Table 1. The content of the nitrogen was 4.62%, 4.33%, 6.25%, and 3.89% for NEHB, NEMB, NEWB, and NEASB, respectively. The content of carbon in different types of nitrogen-enriched biochar was 68.36%, 52.00%, 71.57%, and 88.68% for NEHB biochar, NEMB, NEWB, and NEASB, respectively. Sulfur content in nitrogen-enriched biochar was 0.69%, 0.57%, 0.69%, and 0.59% for NEHB, NEMB, NEWB, and NEASB, respectively. Hydrogen content in nitrogen-enriched biochar was 1.80%, 1.36%, 2.67%, and 1.45% for NEHB, NEMB, NEWB, and NEASB, respectively. Compared to the original biochar, the content of carbon, sulfur, and hydrogen decreased after the modification of biochar. The pH of the original biochar was 8.45, 8.86, 7.54, and 8.37 for HB, MB, WB, and ASB, respectively. On the other hand, the pH of nitrogen-enriched biochar was 5.17, 5.57, 4.42, and 4.52 for NEHB, NEMB, NEWB, and NEASB, respectively. These findings indicate that the type of feedstock played a significant role in the chemical characteristics of both unmodified and modified biochar used in this study.

### Effect of nitrogen-enriched Biochar on some soil chemical properties

#### Soil electrical conductivity

The applications of HB, NEHB, MB, NEMB, and ASB to calcareous sandy soil significantly increased (*p* ≤ 0.01) electrical conductivity (EC) values compared to the control treatment after the harvesting of the spinach plant (Table 3). However, the WB, NEWB, and NEASB applications to the soil under study led to significantly decreased EC values compared to the control treatment. The EC values in calcareous sandy soil increased from 0.92 dS m^− 1^ for the control treatment to 1.22, 1.17, 1.06, 1.23, and 1.05 dS m^− 1^ for HB, NEHB, MB, NEMB, and ASB treatments, respectively. On the other hand, the values of EC decreased from 0.92 dS m^− 1^ (control treatment) to 0.47, 0.57, and 0.74 dS m^− 1^ for WB, NEWB, and NEASB treatments, respectively. Moreover, the relative increase in EC values over the control treatment was 32.61%, 27.50%, 15.22%, 33.37%, and 13.80% for HB, NEHB, MB, NEMB, and ASB treatments, respectively. However, the relative decrease in EC values compared to the control treatment was 49.24%, 38.04%, and 19.57% for WB, NEWB, and NEASB treatments, respectively. The lowest value of EC was observed when the WB treatment was added. However, the highest values were noticed in the HB and NEMB treatments (Table 3).

**Table 3:** Effects of different types of Biochar and nitrogen-enriched Biochar on electrical conductivity, available ammonium, available nitrate, and available potassium after harvesting spinach plant growing in calcareous sandy soil. Values represented are means ± standard error (*n* = 3 analytical replicates)

Treatment	EC (1:1) (dS m^− 1^)	Available NH_4_ (mg kg^− 1^)	Available NO_3_ (mg kg^− 1^)	Available *P* (mg kg^− 1^)	Available K (mmol kg^− 1^)
CK	0.92 ± 0.04c	79.80 ± 2.10a	119.70 ± 3.64b	5.57 ± 0.15a	4.70 ± 0.05e
HB	1.22 ± 0.01a	73.50 ± 2.10ab	126.00 ± 000ab	6.53 ± 0.52a	8.24 ± 0.11a
NEHB	1.17 ± 0.01a	60.90 ± 2.10cde	157.50 ± 18.19ab	5.54 ± 0.35a	5.96 ± 0.11 cd
MB	1.06 ± 0.02b	75.60 ± 0.00ab	126.00 ± 3.64ab	6.25 ± 0.43a	8.41 ± 0.03a
NEMB	1.23 ± 0.01a	50.40 ± 0.00e	170.10 ± 7.27a	7.16 ± 0.38a	5.52 ± 0.09d
WB	0.47 ± 0.00e	56.70 ± 3.64de	121.80 ± 5.56b	6.74 ± 0.65a	6.41 ± 0.14bc
NEWB	0.57 ± 0.01e	69.30 ± 0.00abc	170.10 ± 7.27a	7.59 ± 0.38a	4.87 ± 0.05e
ASB	1.05 ± 0.03b	65.10 ± 2.10bcd	138.60 ± 3.64ab	6.03 ± 0.67a	6.66 ± 0.08b
NEASB	0.74 ± 0.01d	58.80 ± 2.10cde	161.70 ± 2.10ab	6.93 ± 0.57a	5.94 ± 0.11 cd
Coefficient of variation (%)	3.11	5.23	9.10	12.85	2.54

Different lowercase letters in each column showed significant differences between treatments using Tukey’s Honestly Significant Difference test at *P* < 0.01. CK: control (unamended soil); HB: halfa grass biochar; NEHB: nitrogen-enriched halfa grass biochar; MB: marvel grass biochar; NEMB: nitrogen-enriched marvel grass biochar; WB: willow branches biochar; NEWB: nitrogen-enriched willow branches biochar; ASB: apple of Sodom biochar; NEASB: nitrogen-enriched apple of Sodom biochar. EC: electrical conductivity; NH_4_: ammonium; NO_3_: nitrate; P: phosphorus; K: potassium

### Soil nutrient availability

Ammonium availability in calcareous sandy soil decreased significantly after the harvesting of spinach plant with the applications of NEHB, NEMB, NEMB, WB, CB, and NEASB treatments compared with the control treatment (Table 3). The concentrations of available ammonium decreased from 79.80 mg kg^− 1^ (control treatment) to 73.50, 60.90, 75.60, 50.40, 56.70, 69.30, 65.10, and 58.80 mg kg^− 1^ for HB, NEHB, MB, NEMB, WB, NEWB, ASB, and NEASB treatments, respectively. Therefore, the relative decrease in the available ammonium over the control treatment was 7.89%, 23.68%, 5.26%, 36.84%, 28.95%, 13.16%, 18.42%, and 26.32% for HB, NEHB, MB, NEMB, WB, NEWB, ASB, and NEASB treatments, respectively. The lowest concentration of available ammonium was observed in the soil under applying NEMB treatments. The effectiveness of the treatments in this study on the decreasing available ammonium was in the order of control >  MB >  HB >  NEWB >  ASB >  NEHB >  NEASB >  WB > NEMB (Table 3). Nitrate availability increased significantly after harvesting spinach plant with the applications of NEMB and NEWB treatments compared to the control treatment (Table 3). Available nitrate concentrations increased from 119.70 mg kg^− 1^ (control treatment) to 126.00, 157.50, 126.00, 170.10, 121.80, 170.10, 138.60, and 161.70 mg kg^− 1^ for HB, NEHB, MB, NEMB, WB, NEWB, ASB, and NEASB treatments, respectively. The applied biochar treatments increased available nitrate over the control by more pronounced percentages, reaching 5.26%, 31.58%, 5.26%, 42.11%, 1.75%, 42.11%, 15.79%, and 35.09% for HB, NEHB, MB, NEMB, WB, NEWB, ASB, and NEASB treatments, respectively. The highest concentrations of available nitrate were observed in the soil when NEMB and NEWB treatments were applied. The effectiveness of the treatments in this study on the available nitrate enhancement was in the order of NEMB =  NEWB > NEASB >  NEHB >  ASB >  HB =  MB >  WB > control (Table 3). The applications of all treatments to the soil don’t have any significant effect on the available phosphorus after the harvesting of the spinach plant (Table 3). Compared to the control treatment, applying HB, NEHB, MB, NEMB, WB, ASB, and NEASB treatments led to a significant increase in available potassium in calcareous sandy soil. The content of available K increased from 4.70 mmol kg^− 1^ for control treatment to 8.24, 5.96, 8.41, 5.52, 6.41, 6.66, 10.80, and 5.94 mmol kg^− 1^ for HB, NEHB, MB, NEMB, WB, ASB, and NEASB treatments, respectively (Table 3). The relative increase in the available potassium over the control treatment was 75.45%, 26.81%, 78.94%, 17.45%, 36.45%, 3.74%, 41.70%, and 26.45% for HB, NEHB, MB, NEMB, WB, NEWB, ASB, and NEASB treatments, respectively. The highest value of available potassium in the soil was observed in the MB treatment. The effectiveness of the treatments in this study on the available potassium improvement was in the order of MB >  HB >  ASB >  WB >  NEHB >  NEASB >  NEWB > control (Table 3).

### Effect of nitrogen-enriched biochar on growth parameters of the spinach plant

#### Chlorophyll and height of the spinach plant

Compared to the control treatment, applying HB, NEHB, MB, NEMB, NEWB, ASB, and NEASB significantly increased the chlorophyll value of spinach plant grown in calcareous sandy soil. The chlorophyll value increased from 24.85 SPAD (control treatment) to 35.40, 59.71, 31.34, 61.05, 26.03, 61.48, 31.07, and 60.84 SPAD for the HB, NEHB, MB, NEMB, WB, NEWB, ASB, and NEASB treatments, respectively (Table 4). The highest values of chlorophyll in the spinach plant were observed in the NEMB and NEWB treatments. The effectiveness of the treatments in this study on the chlorophyll in spinach plant improvement was in the order of NEWB >  NEMB >  NEASB >  NEHB >  HB >  ASB >  WB > control (Table 4). The results of this study suggest that the modified biochar treatments showed higher chlorophyll values ​​than the unmodified biochar treatments and the control treatment. Adding NEHB, NEMB, NEWB, and NEASB to calcareous sandy soil led to a significant increase in the height of the spinach plant (Table 3). The height of the spinach plant increased from 7.77 cm for the control treatment to 8.23, 13.05, 7.88, 12.57, 11.93, and 11.85 cm for the HB, NEHB, MB, NEMB, NEWB, and NEASB treatments, respectively (Table 4).

**Table 4:** Effects of different types of Biochar and nitrogen-enriched Biochar on chlorophyll, plant height, fresh shoot, and dry shoot of spinach plant growing in calcareous sandy soil. Values represented are means ± standard error (*n* = 3 analytical replicates)

Treatment	Chlorophyll (SPAD)	Plant height (cm)	Fresh shoot (g pot^− 1^)	Dry shoot (g pot^− 1^)
CK	24.85 ± 0.39e	7.77 ± 0.25 cd	9.36 ± 0.74d	1.75 ± 0.08d
HB	35.40 ± 0.62c	8.23 ± 0.05c	15.19 ± 1.03c	2.69 ± 0.11c
NEHB	59.71 ± 0.10b	13.05 ± 0.03a	32.31 ± 0.45a	6.60 ± 0.05a
MB	31.34 ± 0.216d	7.88 ± 0.22 cd	8.03 ± 0.42d	1.45 ± 0.07d
NEMB	61.05 ± 0.11ab	12.57 ± 0.10ab	31.52 ± 0.95ab	6.75 ± 0.12a
WB	26.03 ± 0.04e	7.13 ± 0.27d	9.09 ± 0.25d	1.80 ± 0.05d
NEWB	61.48 ± 0.07a	11.93 ± 0.01b	35.13 ± 0.11a	7.05 ± 0.03a
ASB	31.07 ± 0.21d	7.32 ± 0.16 cd	8.02 ± 1.07d	1.58 ± 0.22d
NEASB	60.84 ± 0.01ab	11.85 ± 0.06b	27.69 ± 0.75b	5.72 ± 0.12b
Coefficient of variation (%)	1.07	2.82	6.37	4.68

Different lowercase letters in each column showed significant differences between treatments using Tukey’s Honestly Significant Difference test at *P* < 0.01. CK: control (unamended soil); HB: halfa grass biochar; NEHB: nitrogen-enriched halfa grass biochar; MB: marvel grass biochar; NEMB: nitrogen-enriched marvel grass biochar; WB: willow branches biochar; NEWB: nitrogen-enriched willow branches biochar; ASB: apple of Sodom biochar; NEASB: nitrogen-enriched apple of Sodom biochar

### The fresh and dry shoot of the spinach plant

The applications of HB, NEHB, NEMB, NEWB, and NEASB treatments to calcareous sandy soil significantly increased the fresh and dry shoot of the spinach plant compared to the control treatment (Table 4). The fresh shoot of the spinach plant increased from 9.36 g pot^− 1^ for the control treatment to 15.19, 32.31, 31.52, 35.13, and 27.69 g pot^− 1^ for HB, NEHB, NEMB, NEWB, and NEASB treatments, respectively. The application of HB, NEHB, NEMB, NEWB, and NEASB treatments increased the fresh shoot of spinach plant over the control by 62.34%, 245.27%, 236.86%, 275.41%, and 195.90%, respectively (Table 4). On the other hand, the application of MB, WB, and ASB treatments decreased the fresh shoot of the spinach plant over the control by 14.18%, 2.85%, and 14.29%, respectively. The effectiveness of treatments in improving the fresh shoot of the spinach plant was in the order of NEWB > NEHB > NEMB > NEASB > HB > control > WB > MB > ASB. The dry shoot of the spinach plant increased from 1.75 g pot^− 1^ (control treatment) to 2.69, 6.60, 6.75, 1.80, 7.05, and 5.72 g pot^− 1^ for HB, NEHB, NEMB, WB, NEWB, and NEASB treatments, respectively. Moreover, the application of HB, NEHB, NEMB, WB, NEWB, and NEASB treatments increased the dry shoot of the spinach plant over the control by 53.54%, 276.97%, 285.54%, 2.86%, 302.69%, and 226.86%, respectively (Table 4). On the other hand, adding MB and ASB treatments decreased the dry shoot of the spinach plant over the control by 17.14% and 9.89%, respectively. The treatments used in this soil showed improvements in the dry shoot of the spinach plant in the order of in the order of NEWB >  NEMB >  NEHB >  NEASB >  HB >  WB >  control >  ASB > MB. According to the results obtained from this study, the highest values of fresh and dry shoot of the spinach plant were noticed in the NEWB applications. The lowest values of fresh and dry shoot of spinach in this study were recorded for the CB treatment (Table 4).

### Effect of nitrogen-enriched biochar on the concentrations of nutrients in the spinach plant

The applications of NEHB, NEMB, NEWB, and NEASB treatments to calcareous sandy soil significantly increased nitrogen content in spinach plant compared with the control treatment (Table 5). However, applying HB, MB, and ASB treatments to calcareous sandy soil resulted in a non-significant decrease in nitrogen content in the spinach plant compared with the control treatment (Table 5). Nitrogen content in spinach plant increased from 10.56 g kg^− 1^ for the control treatment to 17.94, 17.50, 17.72, and 16.82 g kg^− 1^ for NEHB, NEMB, NEWB, and NEASB treatments, respectively. However, nitrogen content in spinach plant decreased from 10.56 g kg^− 1^ for the control treatment to 8.65, 8.68, 9.55, and 9.84 g kg^− 1^ for the HB, MB, WB, and ASB treatments, respectively. The effectiveness of the treatments in this study on improving nitrogen content in spinach plant was in the order of NEHB >  NEWB >  NEMB >  NEASB >  control >  ASB >  WB >  MB >  HB (Table 5). According to the results obtained from this study, the highest values of nitrogen content in spinach plant were observed in all treatments of nitrogen-enriched biochar. The lowest values of nitrogen content in spinach plant in this study were recorded for all treatments of unmodified biochar. Compared to the control treatment, a significant increase in phosphorus content in spinach plant was noticed under additions of HB, MB, WB, and ASB treatments (Table 5). But applying NEHB, NEMB, NEWB, and NEASB to the soil resulted in a significant decrease in phosphorus content in the spinach plant compared to the control treatment (Table 5). Phosphorus content in the spinach plant increased from 1.35 g kg^− 1^ for the control treatment to 2.52, 3.46, 3.40, and 2.35 g kg^− 1^ for HB, MB, WB, and ASB treatments, respectively. However, phosphorus content in the spinach plant decreased from 1.35 g kg^− 1^ for the control treatment to 0.69, 0.75, 0.77, and 0.76 g kg^− 1^ for NEHB, NEMB, NEWB, and NEASB treatments, respectively. According to the results obtained from this study, the highest values of phosphorus content in spinach plant were observed in all treatments of unmodified biochar. The lowest values of phosphorus content in the spinach plant in this study were recorded for nitrogen-enriched biochar treatments. In our study, the effectiveness of the treatments on improving phosphorus content in spinach plant was in the order of MB >  WB >  HB >  ASB >  control >  NEWB >  NEASB >  NEMB > NEHB (Table 5). A significant increase in potassium content occurred in spinach plant under additions of HB, MB, WB, and ASB treatments compared to the control treatment (Table 5). However, applying NEHB, NEMB, NEWB, and NEASB treatments to the soil resulted in a significant decrease in potassium content in spinach plant compared to the control treatment (Table 5). Potassium content in the spinach plant increased from 57.42 g kg^− 1^ for the control treatment to 77.32, 89.92, 73.65, and 69.35 g kg^− 1^ for HB, MB, WB, and ASB treatments, respectively. However, potassium content in the spinach plant decreased from 57.42 g kg^− 1^ for the control treatment to 45.41, 44.82, 41.13, and 45.49 g kg^− 1^ for NEHB, NEMB, NEWB, and NEASB treatments, respectively. According to the results obtained from this study, the highest values of potassium content in spinach plant were observed in all treatments of unmodified biochar. The lowest values of phosphorus content in the spinach plant in this study were recorded for nitrogen-enriched biochar treatments. In our study, the effectiveness of the treatments on improving phosphorus content in spinach plant was in the order of MB > HB > WB > ASB > control > NEASB > NEHB > NEMB > NEWB (Table 5).

**Table 5:** Effects of different types of Biochar and nitrogen-enriched Biochar on nutrient concentrations in spinach plant grown in calcareous sandy soil. Values represented are means ± standard error (*n* = 3 analytical replicates)

Treatment	Nutrient concentrations (g kg^− 1^)		
	Nitrogen	Phosphorus	Potassium
CK	10.56 ± 0.26b	1.35 ± 0.15c	57.42 ± 0.54d
HB	8.68 ± 0.33b	2.52 ± 0.04b	77.32 ± 1.66b
NEHB	17.94 ± 0.40a	0.69 ± 0.06d	45.41 ± 0.65e
MB	8.68 ± 0.25b	3.46 ± 0.03a	89.92 ± 2.35a
NEMB	17.50 ± 0.19a	0.75 ± 0.02 cd	44.81 ± 1.16e
WB	9.55 ± 0.22b	3.40 ± 0.18a	73.65 ± 0.60bc
NEWB	17.72 ± 0.52a	0.77 ± 0.03 cd	41.13 ± 0.15e
ASB	9.84 ± 0.36b	2.35 ± 0.17b	69.35 ± 1.42c
NEASB	16.82 ± 0.06a	0.76 ± 0.06 cd	45.48 ± 0.64e
Coefficient of variation (%)	4.20	10.03	3.46

Different lowercase letters in each column showed significant differences between treatments using Tukey’s Honestly Significant Difference test at *P* < 0.01. CK: control (unamended soil); HB: halfa grass biochar; NEHB: nitrogen-enriched halfa grass biochar; MB: marvel grass biochar; NEMB: nitrogen-enriched marvel grass biochar; WB: willow branches biochar; NEWB: nitrogen-enriched willow branches biochar; ASB: apple of Sodom biochar; NEASB: nitrogen-enriched apple of Sodom biochar

### Effect of nitrogen-enriched biochar on the nutrient uptake by the spinach plant

Nitrogen uptake by the spinach plant significantly increased, resulting in the addition of NEHB, NEMB, NEWB, and NEASB treatments to calcareous sandy soil compared to the control treatment. The content of nitrogen uptake increased from 18.51 mg pot^− 1^ for the control treatment to 23.37, 118.28, 118.12, 124.87, and 96.21 mg pot^− 1^ for HB, NEHB, NEMB, NEWB, ASB, and NEASB treatments, respectively. The highest content of nitrogen uptake by the spinach plant was observed after applying the NEWB treatment (Table 6). The lowest content of nitrogen uptake in the spinach plant occurred at the MB treatment. In the present study, the effectiveness of the treatments on improving nitrogen uptake in the spinach plant was in the order of NEWB >  NEHB >  NEMB >  NEASB >  HB >  control >  WB >  ASB > MB (Table 6). The relative increase in the nitrogen uptake by the spinach plant over the control treatment was 26.27%, 538.97%, 538.18%, 574.61%, and 419.77% for HB, NEHB, NEMB, NEWB, and NEASB treatments, respectively. On the other hand, applying MB, WB, and ASB treatments decreased nitrogen uptake by the spinach plant over the control by 32.18%, 7.24%, and 17.03%, respectively (Table 6). Phosphorus uptake by spinach plant was significantly enhanced by adding HB, MB, NEMB, WB, and NEWB treatments to calcareous sandy soil compared to the control treatment (Table 6). Phosphorus uptake content increased from 2.36 mg pot^− 1^ (control treatment) to 6.76, 4.54, 5.01, 5.05, 6.15, 5.44, 3.77, and 4.38 mg pot^− 1^ for HB, NEHB, MB, NEMB, WB, NEWB, ASB, and NEASB treatments, respectively. The highest content of phosphorus uptake was noticed in HB treatment. The treatments used in this soil showed improvements in phosphorus uptake in the order of HB >  WB >  NEWB >  NEMB >  MB >  NEHB >  NEASB >  ASB > control (Table 6). Phosphorus uptake content in spinach plant under applying NEHB and NEWB was lower than the original biochar of HB and WB. The relative increase in the phosphorus uptake by the spinach plant over the control treatment was 186.81%, 92.32%, 112.69%, 114.13%, 160.92%, 130.93%, 60.08%, and 85.70% for HB, NEHB, MB, NEMB, WB, NEWB, ASB, and NEASB treatments, respectively. Compared to the control treatment, applying HB, NEHB, NEMB, NEWB, and NEASB treatments significantly increased potassium uptake of spinach plant grown in calcareous sandy soil (Table 6). Potassium uptake increased from 100.45 mg pot^− 1^ (control) to 207.95, 299.54, 130.55, 302.46, 132.51, 289.77, 108.85, and 260.10 mg pot^− 1^ for HB, NEHB, MB, NEMB, WB, NEWB, ASB, and NEASB treatments, respectively. The highest content of potassium uptake by the spinach plant was noticed after adding NEMB treatment. The treatments can be ranked in terms of their effectiveness in enhancing potassium uptake as follows: NEMB >  NEHB >  NEWB >  NEASB >  HB >  WB >  MB >  ASB > control (Table 6). The relative increase in the potassium uptake by the spinach plant over the control treatment was 107.03%, 198.21%, 29.97%, 201.11%, 31.92%, 188.48%, 8.37%, and 158.94% for HB, NEHB, MB, NEMB, WB, NEWB, ASB, and NEASB treatments, respectively.

### Effect of nitrogen-enriched biochar on nitrogen use efficiency of the spinach plant

The nitrogen use efficiency (NUE) of spinach plant significantly improved as a result of applying different types of nitrogen-enriched biochar in calcareous sandy soil compared to unmodified biochar treatments (Table 6). The results indicated that the lowest value of NUE of the spinach plant was found at applying ASB treatment. However, the highest value of NUE of the spinach plant was observed after adding the NEWB treatment. The values of NUE of the spinach plant were 205.53, 189.03, 184.44, 162.00, 88.88, 53.19, 46.99, and 46.93 mg shoot mg^− 1^ N for NEWB, NEHB, NEMB, NEASB, HB, WB, MB, and ASB treatments, respectively. These treatments can be ranked in the order of NEWB >  NEHB >  NEMB >  NEASB >  HB >  WB >  MB > ASB (Table 6). The NUE values of the spinach plant under applying all types of nitrogen-enriched biochar were higher than all types of the original biochar.

**Table 6:** Effects of different types of Biochar and nitrogen-enriched Biochar on nutrient uptake and nitrogen use efficiency of spinach plant grown in calcareous sandy soil. Values represented are means ± standard error (*n* = 3 analytical replicates)

Treatment	Nutrient uptake (g pot^− 1^)			NUE (mg yield mg^− 1^ *N*)
	Nitrogen	Phosphorus	Potassium	
CK	18.51 ± 1.18c	2.36 ± 0.24c	100.45 ± 3.89c	–
HB	23.37 ± 1.78c	6.76 ± 0.26a	207.95 ± 12.72b	88.88 ± 6.01c
NEHB	118.28 ± 2.07a	4.54 ± 0.43abc	299.54 ± 6.50a	189.03 ± 2.65a
MB	12.55 ± 0.24c	5.01 ± 0.20ab	130.55 ± 8.62c	46.99 ± 2.43d
NEMB	118.12 ± 2.94a	5.05 ± 0.22ab	302.46 ± 10.86a	184.44 ± 5.54ab
WB	17.17 ± 0.26c	6.15 ± 0.50ab	132.51 ± 2.87c	53.19 ± 1.45d
NEWB	124.87 ± 4.01a	5.44 ± 0.20ab	289.77 ± 2.13a	205.53 ± 0.62a
ASB	15.36 ± 1.64c	3.77 ± 0.79bc	108.85 ± 13.99c	46.93 ± 6.28d
NEASB	96.21 ± 2.30b	4.38 ± 0.39abc	260.10 ± 4.69a	162.00 ± 4.41b
Coefficient of variation (%)	6.16	14.43	7.19	5.96

Different lowercase letters in each column showed significant differences between treatments using Tukey’s Honestly Significant Difference test at *P* < 0.01. CK: control (unamended soil); HB: halfa grass biochar; NEHB: nitrogen-enriched halfa grass biochar; MB: marvel grass biochar; NEMB: nitrogen-enriched marvel grass biochar; WB: willow branches biochar; NEWB: nitrogen-enriched willow branches biochar; ASB: apple of Sodom biochar; NEASB: nitrogen-enriched apple of Sodom biochar. NUE: nitrogen use efficiency

## Discussion

According to a previous study, the carbon content in different types of biochar produced at 400 °C was 64.18%, 63.36%, 63.74%, and 56.42% for wheat straw biochar, corn straw biochar, rape straw biochar, and rice straw biochar, respectively. The content of nitrogen in biochar produced at 400 °C was 1.36%, 2.52%, 0.98%, and 1.99% for wheat straw biochar, corn straw biochar, rape straw biochar, and rice straw biochar, respectively. Sulfur content in biochar produced at 400 °C was 0.47%, 0.51%, 0.68%, and 0.44% for wheat straw biochar, corn straw biochar, rape straw biochar, and rice straw biochar, respectively. Hydrogen content in biochar produced at 400 °C was 1.78%, 1.96%, 1.91%, and 1.35% for wheat straw biochar, corn straw biochar, rape straw biochar, and rice straw biochar, respectively. Moreover, the pH values for different types of biochar produced at 400 °C were 9.06%, 9.83%, 9.18%, and 9.33% for wheat straw biochar, corn straw biochar, rape straw biochar, and rice straw biochar, respectively [40]. Another study reported that preparing nitrogen-enriched biochar through nitric acid treatment of the original biochar was characterized by C (58.14%), N (4.99%), H (3.10%), O (32.70%), and a pH of 4.8 [41]. The pH of biochar decreases after modification with nitric acid compared to the original biochar [42]. Biochar treated with nitric acid exhibited a decrease in carbon and hydrogen content, and an increase in oxygen and nitrogen content. Carbon reduction, as well as nitrogen and oxygen increase, may be attributed to the destruction of pore walls, the formation of aromatic compounds containing nitrogen, and the creation of carbon-oxygen functional groups on the surface [43]. Oxygen functional groups on the surface of biochar were responsible for an important part of nitrogen retention. In addition to incorporating external nitrogen sources, nitric acid oxidation inherently increased the nitrogen content of the biochar by generating nitro groups and pyrrolic-type nitrogen [42]. The presence of water-soluble, hydrolyzable, and non-hydrolyzable nitrogen, as well as some of the characteristics mentioned previously, indicates that the nitrogen-enriched biochar may provide nitrogen that would be progressively available for plants, acting as a slow-release fertilizer [42].

Applying nitrogen-enriched biochar to the soil led to a significant increase in electrical conductivity in the soil solution [44]. This increase may be attributed to treating biochar with nitric acid after pyrolysis, which leads to an increase in electrical conductivity. This means that acid modification increased the total water-soluble nutrient content in the biochar samples [45]. Several studies reported that adding biochar to calcareous sandy soil significantly increases the electrical conductivity of the soil solution [46, 47]. The increases in electrical conductivity in the soil solution are attributed to the biochar is rich in higher soluble salts of alkali and alkaline earth cations [48]. Incorporating sorghum panicles biochar and wood chips biochar with urea fertilizer in saline sandy soil significantly decreased electrical conductivity compared to the unamended soil [49]. This is attributed to the retention of sodium on the biochar surfaces and the capture of salts in biochar pores [50]. Adding nitrogen-enriched biochar to the soils improved total nitrogen concentrations [51]. The application of nitrogen-enriched biochar significantly increased nitrate content in the soil [52]. Co-application of biochar with nitrogen fertilizer in calcareous sandy soil resulted in a significant increase in bioavailable nitrogen compared to the unamended soil [53] because the incorporation of biochar with nitrogen fertilizer into the sandy soil decreased the concentrations of nitrate and ammonium leached, which in turn led to an increase in the available nitrogen [54, 55]. The results of the current study are consistent with previous findings, which reported higher concentrations of available nitrate than ammonium in soils amended with biochar, likely due to increased activity of the nitrification process [56]. Moreover, applying biochar to the soil decreased ammonium concentration, which can be attributed to enhanced ammonium assimilation and increased nitrification [57]. Biochar application to the Soil played an important role in increasing the adsorption of ammonium and nitrate ions, which decreased leaching of ammonia and nitrate from the amended soil [58] and reduced ammonia volatilization from the soil [59]. Biochar application affects the bioavailability of nitrogen in soils through several mechanisms, such as mineralization, immobilization, denitrification, plant uptake, fixation, adsorption, volatilization, and leaching [60]. A previous study reported that nitrogen enrichment of biochar promoted interactions between nitrogen and functional groups on biochar surfaces, thereby slowing its release and simultaneously reducing ammonia volatilization more effectively than modified biochar [22]. Consistent with the current results, the applications of nitrogen-enriched biochar to sandy loam caused a significant increase in potassium availability compared to the unamended soil 61 [61]. Generally, several studies found that adding biochar caused an increase in potassium availability in calcareous sandy soil [

### Pot experiment

62, 63]. These increases in available potassium in the soil are attributed to the fact that biochar contains considerable amounts of soluble and exchangeable alkali and alkaline earth cations (Ca^2+^, Mg^2+^, K^+^, and Na^+^), which can be released into the soil, helping enhance soil fertility [64]. The decrease in available potassium in the soil treated with nitrogen-enriched biochar compared to the original biochar may be attributed to the increased amount of potassium uptake by the spinach plant. These results are consistent with the findings reported by Mengel and Kirkby [65], who suggested that the nutrients were removed from the soil solution by the uptake of crops. Modification of biochar caused an increase in surface area, cation exchange capacity, and porous structure [66, 67]. Chemical modifications increased potassium binding sites in the modified biochar. This was substantiated by the presence of minerals containing potassium, such as taranakite and feldspar [68]. Moreover, the strengthening of soil potassium retention resulting from the addition of biochar may be due to its structural properties, such as its porous structure, large surface area, and negative surface charge [69]. Biochar modification is particularly suitable for nutrient-poor soils, providing a high adsorption capacity [10]. The addition of nitrogen-enriched biochar can improve soil properties more than synthetic fertilizer, which in turn leads to improving soil quality indicators [70]. Improvement of soil properties and fertility through biochar application may subsequently lead to enhancing crop yield and nitrogen use efficiency.

The application of nitrogen-enriched biochar to the sandy soil significantly enhanced plant height and dry matter of the maize plant [71]. Moreover, adding nitrogen-enriched biochar to the soil enhanced crop yield and total biomass of rice plant [72]. These positive responses can be explained by the superior performance of nitrogen-enriched biochar, which outperforms the modified biochar. This superiority is attributed to the slower nitrogen release and mineralization rates of the nitrogen-enriched biochar compared to the modified biochar [22]. Applying nitrogen-enriched biochar promoted cotton growth, chlorophyll content, physiological responses, and yield-contributing traits. Also, a significant increase in nitrogen uptake was observed in wheat plant under the application of nitrogen-enriched biochar in the soil [52]. The application of nutrient-enriched biochar to the soil increased the uptake of nitrogen, phosphorus, and potassium by the canola plant compared to the control treatment [60]. On the other hand, a previous study showed that co-applying two types of biochar with ammonium nitrate decreased phosphorus content in Swiss chard leaves compared to biochar alone, although it increased phosphorus content in the soil. This is attributed to the occurrence of phosphorus sorption by biochar, which in turn reduced phosphorus availability for plant uptake [28]. Additionally, some studies have found that after adding biochar to soil, phosphorus ions may precipitate with calcium and magnesium ions released from the biochar or form co-precipitates with mineral complexes on the biochar surface [73, 74]. This finding is supported by previous research showing that nitrogen application to the soil contributes to a decrease in phosphorus concentration in plant leaves, which may be attributed to the growth dilution effect outpacing the phosphorus uptake capacity [75]. Moreover, the reduction in concentration of phosphorus and potassium in the shoots is caused by the dilution effect caused by the increased dry matter production [76, 77]. Applying biochar alone, as well as co-applying biochar with chemical fertilizers, to the soils significantly improved crop yield compared with unfertilized soil [78]. Nitrogen fertilizer application to the soil significantly increased chlorophyll content, fresh weight, total yield, and inflorescence length of spinach plant [79]. Nitrogen fertilization improves the nutritional status of the spinach plant, which in turn enhances vegetative and root growth, as evidenced by increases in plant height, number of leaves, and dry weight. All these factors enhance the plant’s ability to uptake nutrients and accumulate them in the spinach plant [80]. Biochar application to the soil significantly increased the yield of spinach plant [81]. Biochar application to the soil increased the concentrations of nutrients in the plant by improving nitrogen use efficiency [60, 82]. Co-applying biochar with the soil improved nitrogen availability, nitrogen uptake, and nitrogen use efficiency by the crops [57]. Application of nitrogen-enriched biochar enhances microbial activity in the soil, which contributes to the dissolution of potassium compounds, converting them into forms more readily available for plant uptake [83]. Combining biochar and nitrogen fertilizer applications can significantly reduce nitrogen loss while promoting root growth and nitrogen uptake, all of which contribute to increased nitrogen use efficiency [84]. Adding modified biochar to the soil affects plant growth through many mechanisms, such as improving the physical and chemical properties of the soil, preventing plants from uptake of heavy metals through adsorbing heavy metals on biochar surfaces, increasing the soil content of organic carbon, nitrogen, phosphorus, and potassium, enhancing antioxidant enzyme activity and preventing plant oxidative stress, improving morphology of plant root, and reducing plant uptake of sodium ions and decrease the harmful effects of salt stress on plants [85]. The use of nitrogen-enriched biochar as a slow-release nitrogen fertilizer has the potential to add significant economic and environmental value [86]. The improvement in spinach growth and nutrient uptake observed in the current study can be directly attributed to the enhancement of soil properties following the addition of different types of nitrogen-enriched biochar to calcareous sandy soil. These amendments decreased electrical conductivity and increased the availability of essential nutrients—particularly nitrogen and potassium. The slow and sustained release of nitrogen from the nitrogen-enriched biochar ensured a continuous nutrient supply throughout the growth period. These changes created a more favorable soil environment, which ultimately increased biomass in the spinach plant.

Nutrient use efficiency of a plant indicates the soil’s capacity to supply sufficient nutrient levels and the plant’s ability to uptake and transport within roots and shoot, and remobilize nutrients to other parts of the plant [87]. The co-application of biochar with nitrogen fertilizer enhanced the nitrogen use efficiency of maize growing in acidic soil [88]. The nitrogen use efficiency of the zucchini plant was significantly improved by co-applying biochar with nitrogen fertilizer in calcareous sandy soil [42]. Amending soil with engineered biochar improved the physical properties of soil, decreased nitrogen input, and enhanced nitrogen metabolism, which in turn improved nitrogen use efficiency and crop growth [89]. Increasing nitrogen use efficiency is essential for enhancing crop yield, reducing nitrogen fertilizer demand, and mitigating environmental pollution [90]. Furthermore, improving fertilizer use efficiency reduces nutrient loss in ecosystems and also reduces the cost of adding fertilizer [87]. The slow release of nitrogen can be used very efficiently in agricultural applications, greatly reducing nitrogen losses [91]. Improving nitrogen use efficiency is a key strategy in sustainable farming practices, helping to balance the need for increasing food production with the imperative to protect natural ecosystems [92].

Therefore, this study highlighted that the production of nitrogen-enriched biochar from locally available plant residues for soil amendment represents a promising approach to sustainable agriculture. Nevertheless, the additional benefits associated with nitrogen-enriched biochar application should be taken into account. Beyond its positive effects on soil properties, crop growth, and improving nutrient-use efficiency, the contribution of biochar to carbon sequestration remains a key advantage that contributes substantially to climate change mitigation. Consequently, nitrogen-enriched biochar not only supplies nitrogen efficiently over time but also represents a more economical option compared to expensive conventional chemical fertilizers. The results of this study highlight several directions for future research. Field experiments are required to assess the effectiveness of nitrogen-enriched biochar under different soils and climate conditions and across different plant species. A study of the effect of nitrogen-rich biochar produced from various plant residues on the activity and growth of soil microorganisms under field conditions should be carried out.

## Conclusions

The application of chemical fertilizers undoubtedly improves agricultural production, but it also causes significant environmental problems and substantial economic burdens. Techniques for converting biochar into nitrogen-enriched biochar have made it possible to obtain a product with high nutritional value to enhance soil fertility and plant growth. The potential use of nitrogen-enriched biochar as a slow-release fertilizer is a promising strategy to reduce production costs in sustainable agriculture. Adding all types of nitrogen-enriched biochar led to an increase in nutrient availability in calcareous sandy soil. Applying nitrogen-enriched biochar to calcareous sandy soil promoted spinach growth, biomass yield, and nitrogen use efficiency. Nitrogen-enriched biochar is likely to be an effective alternative to chemical nitrogen fertilizers in calcareous sandy soil, providing a slow and sustained release of nitrogen while potentially reducing overall fertilizer expenses. Most studies on nitrogen-enriched biochar have been conducted in laboratories and greenhouses over a short period. Therefore, future studies should focus on elucidating the mechanisms of the long-term application of nitrogen-enriched biochar on plant growth and soil properties. Another important area for exploration is the potential of nitrogen-enriched biochar to improve the agronomic performance of crops, particularly under field conditions.

## Data Availability

The datasets used or analyzed during the current study are available from the corresponding author upon reasonable request.
